# Infection risk in patients with autoimmune cytopenias and immune dysregulation treated with mycophenolate mofetil and sirolimus

**DOI:** 10.3389/fimmu.2024.1415389

**Published:** 2024-05-30

**Authors:** Mattia Comella, Elena Palmisani, Marcello Mariani, Gianluca Dell’Orso, Maria Licciardello, Maria Carla Giarratana, Luca Arcuri, Sara Pestarino, Alice Grossi, Marina Lanciotti, Giorgia Brucci, Daniela Guardo, Giovanna Russo, Carlo Dufour, Francesca Fioredda, Elio Castagnola, Maurizio Miano

**Affiliations:** ^1^ Haematology Unit, Department of Haematology/Oncology, IRCCS Istituto Giannina Gaslini, Genoa, Italy; ^2^ Pediatric Hematology and Oncology Unit, Department of Clinical and Experimental Medicine, University of Catania, Catania, Italy; ^3^ Infectious Diseases Unit, Department of Pediatrics, IRCCS Istituto Giannina Gaslini, Genova, Italy; ^4^ Genetic and Genomic of Rare Disease Unit, IRCCS Istituto Giannina Gaslini, Genova, Italy; ^5^ Department of Health Sciences (DISSAL), University of Genoa, Genoa, Italy

**Keywords:** mycophenolate, sirolimus, infection, PIRDs, ALPS, ALPS-like, ITP, autoimmune cytopenia

## Abstract

**Introduction:**

Autoimmune cytopenias (AICs) are a group of disorders characterized by immune-mediated destruction of blood cells. In children, they are often secondary to immune dysregulation that may require long-lasting immunosuppression. Mycophenolate mofetil and sirolimus represent two well-tolerated options to treat these disorders, often as a steroid-sparing option. However, no data are available on the infection risk for patients undergoing long-lasting treatments.

**Patients and methods:**

The rate of severe infective events was calculated in episodes per 100 persons/months at risk (p/m/r) documented by the analysis of hospitalization charts between January 2015 and July 2023 of patients treated with mycophenolate mofetil or sirolimus given for isolated AIC or AICs associated with autoimmune lymphoproliferative syndrome (ALPS)/ALPS-like syndromes in two large Italian pediatric hematology units.

**Results:**

From January 2015 to July 2023, 13 out of 96 patients treated with mycophenolate mofetil or sirolimus developed 16 severe infectious events requiring hospitalization. No patients died. Overall infection rate was 0.24 person/*100 months/risk (95% CI 0.09–0.3). Serious infectious events incidence was higher in patients with ALPS-like compared to others (0.42 versus 0.09; *p* = 0.006) and lower in patients who underwent mycophenolate treatment alone compared to those who started sirolimus after mycophenolate failure (0.04 versus 0.29, *p* = 0.03). Considering only patients who started treatment at the beginning of study period, overall cumulative hazard was 18.6% at 60 months (95% CI 3.4–31.4) with higher risk of infectious events after 5 years in ALPS-like patients (26.1%; 95% CI 3.2–43.5) compared to other AICs (4%; 95% CI 0–11.4; *p* = 0.041).

**Discussion:**

To the best of our knowledge, this is the first study to describe the infectious risk related to mycophenolate and sirolimus chronic treatment in patients with AICs and immune dysregulation. Our data highlight that infection rate is very low and mainly related to the underlying hematological condition.

**Conclusions:**

Mycophenolate and sirolimus represent a safe immunosuppressive therapy in AICs and immune dysregulation syndromes.

## Introduction

Autoimmune cytopenias (AICs) are a group of heterogeneous disorders characterized by immune-mediated destruction of one or more hematopoietic lineage cells, which include immune thrombocytopenic purpura (ITP), autoimmune hemolytic anemia (AIHA), autoimmune neutropenia (AIN), and Evans syndrome (ES) (1–9).

AICs may be isolated (idiopathic) or secondary to (i) autoimmune diseases, (ii) immunodeficiencies, (iii) tumors, (iv) medications, or (v) infections. In children, primary immunoregulatory disorders (PIRDs), a group of diseases characterized by the presence of autoimmunity and benign lymphoproliferation, represent frequent underlying conditions (8). Autoimmune lymphoproliferative syndrome (ALPS), a monogenic disorder secondary to defects of FAS-dependent apoptosis, is one of the first described examples of predisposing condition to AIC (10). Other novel PIRDs, often reported as ALPS-like disorders, secondary to the impairment of other molecular pathways, have been increasingly reported over the years (11). The clinical phenotype of these disorders is usually very heterogeneous. The intrinsic predisposition to infections is variable and usually related to the underlying molecular defect, being virtually absent in patients with ALPS (12) and more pronounced in most ALPS-like disorders, often characterized by hypogammaglobulinemia and defect of B-cell function.

In most cases, corticosteroids are given as first-choice treatment for AICs and/or PIRDs. However, some patients do not respond or become steroid-dependent and, therefore, at risk of undergoing severe side effects such as endocrine dysfunctions, osteoporosis, and avascular necrosis (3). Therefore, other long-term therapies are often required to control the disease and to reduce the risk of side effects. Mycophenolate mofetil (MMF) and sirolimus (SR) represent two manageable and well-tolerated (1) drugs that have been widely used either in patients undergoing solid organ transplantation or those with AICs and PIRDs. MMF, an inhibitor of inosine monophosphate dehydrogenase in purine synthesis that targets B, T, and NK cells (13), has been shown to be effective in AIHA and immune thrombocytopenia in some retrospective studies on both adult and children (14–16). SR, an inhibitor of the mammalian target of rapamycin (mTOR), has been used for many years (17) in the setting of autoimmune diseases, solid organ transplants, and AIHA (18–20). SR has also been reported as an effective treatment for ALPS patients with or without cytopenia (21) by reducing the count of double-negative T cells (22), by increasing T-regulatory cells (T-regs), and by inducing apoptosis of abnormal lymphocytes. However, no data are available on the risk of infections in the setting of hematological patients under such treatments. The aim of this study is to evaluate the infectious risk of MMF and SR treatments in patients with ALPS, ALPS-like, and AIC referred at two main Italian pediatric hematology centers.

## Patients and methods

Patients diagnosed with ALPS/ALPS-like syndromes with or without AIC and patients with isolated AICs treated with MMF or SR at the Hematology Unit of IRCCS Istituto Giannina Gaslini, Genoa, Italy, and Pediatric Hematology/Oncology Unit of “Policlinico of Catania”, Catania, Italy, from January 2015 to July 2023 were included. Patients fulfilling the revised diagnostic criteria for definitive/probable ALPS were classified as ALPS patients (10). ALPS-like was defined as the presence of AIC associated with one absolute or primary additional criterion for ALPS. A severe infectious event (SIE) was defined as hospitalization and/or the requirement for intravenous antibiotics, independently from microbiological findings. Infection crude rate was calculated in episodes per 100 persons/months at risk (p/m/r) with Poisson 95% confidence interval. Descriptive statistics were produced for demographic and clinical characteristics of patients. Groups were compared with Pearson’s chi-square test (or Fisher’s exact when appropriate) and parametric or nonparametric tests according to data distribution. An SIE-free survival analysis was performed by Kaplan–Meier estimate considering newly diagnosed patients who started immunomodulatory treatment before January 2015. SIE occurring during the first 4 weeks of treatment was excluded, since both drugs are usually active not earlier than 4–6 weeks. Differences between ALPS-like and other AIC groups’ SIE-free survival were assessed by log rank test.

## Results

Between January 2015 and July 2023, a total of 103 patients (51 female patients, 53%) with a median age of 9 years (IQR 10) were identified. Among those, 49/103 (47.6%) were diagnosed after 2015. Seven patients were lost at follow-up after diagnosis. The remaining 96 were treated with MMF (35, 36%) and SR (31, 32%) alone or with SR after MMF failure (30, 31%). Thirty (31%) out of 96 patients were affected with isolated AIC, being ITP in all cases. The remaining 19 (20%) and 47 (49%) suffered from ALPS and ALPS-like syndromes, respectively. MMF and SR were given for a median of 31 months (IQR 71 and 43, respectively). Overall observation time was 6,620 months. Patients who underwent MMF or SR treatments alone were followed for a total of 2,494 and 2,091 months, respectively, while patients with MMF-SR sequential treatment had 2,035 months of total follow-up.

**Figure 1:**
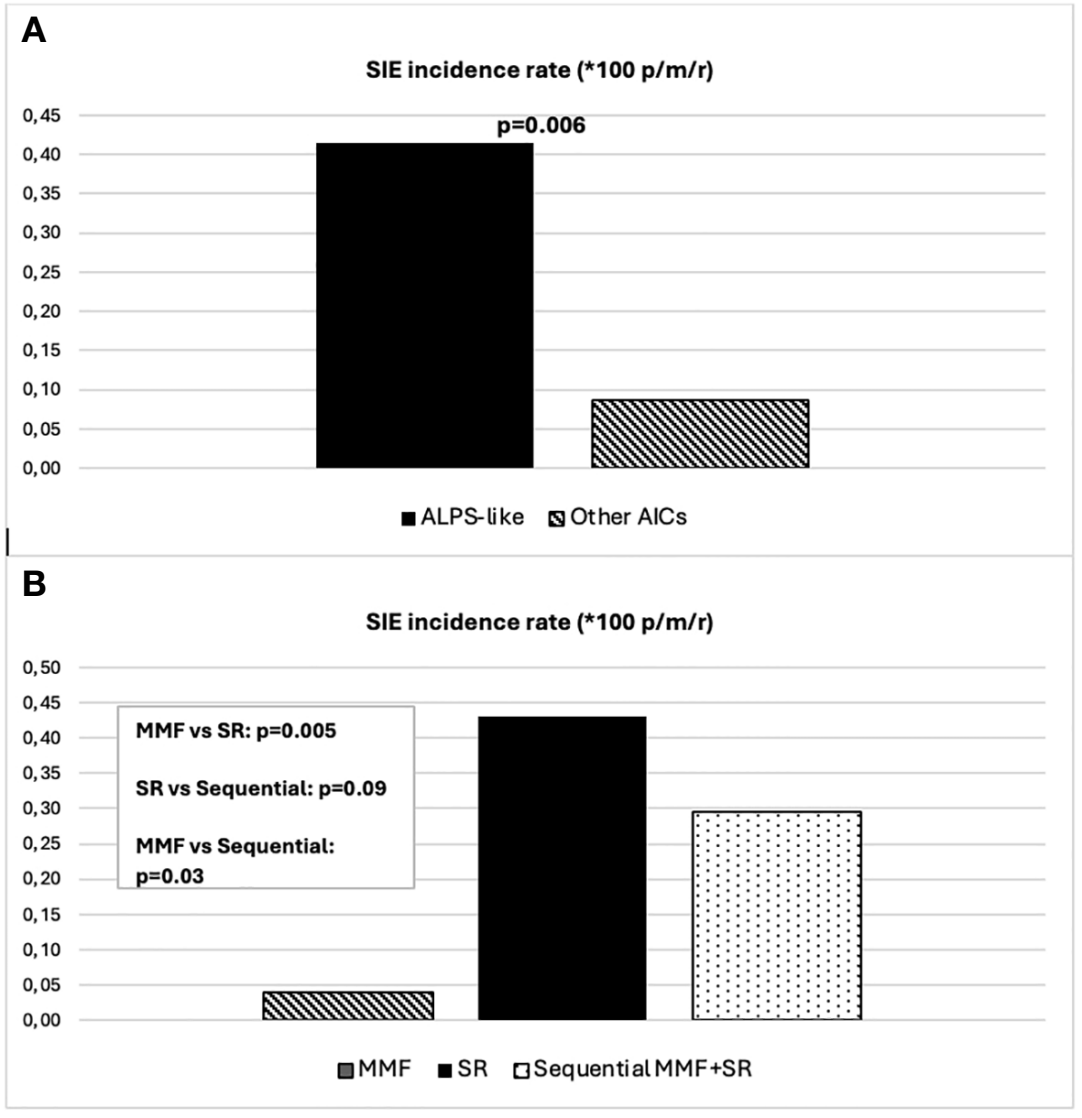
**(A)** SIE incidence rate differences between ALPS-like and other AICs. **(B)** SIE incidence rate differences between treatment schedules.

Forty-five (47%) out of 96 patients developed infections during therapy, mostly mild upper respiratory infections. A total of 16 SIEs were documented in 13/96 patients (13%) and were mostly pneumonias (10). The remaining events consisted of abdominal infections (2), sepsis (2), cryptococcal meningitis (1), and pyelonephritis (1). Clinical features of patients with and without SIEs according to diagnosis and treatment are summarized in **Table 1**. Among patients with at least one SIE, a higher frequency of ALPS-like diagnosis (10/13, 76%) (*p* = 0.038) and history of therapy with both MMF and SR (7/13, 53.8%) (*p* = 0.05) were observed (**Table 1**). Eleven (84.6%) out of 13 patients were under SR treatment at time of SIE. Six (60%) out of 10 ALPS-like patients with SIE were found to have an underlying genetic disorder, which was detected only in 9 out of the remaining 45 (20%) without SIE (*p* = 0.01). **Table 2** shows the clinical features of patients with SIE. Overall, an SIE crude rate of 0.24 *100 p/m/r (95% CI 0.09–0.3) was observed. Diagnosis of ALPS-like was associated with a higher incidence of SIE compared with other diagnosis (0.32 vs. 0.09; *p* = 0.03). MMF treatment was associated with a lower incidence of SIEs compared with patients who underwent only SR or sequential MMF/SR (0.04 *100 p/m/r versus 0.43 and 0.29, respectively; **Figures 1A, B**). Data are summarized in **Table 3**.

**Table 1:** Clinical features of patients with and without SIEs according to diagnosis and treatment.

	Patients with at least 1 SIE (*n* = 13)	No SIEs (*n* = 83)	*p*
*Diagnosis*	*n* (%)	*n* (%)	0.038
ALPS-like	10 (76.9%)	37 (44.6%)	
Other			
*ITP*	1 (7.7%)	29 (34.9%)	
*ALPS*	2 (15.4%)	17 (20.5%)	
***Therapy during follow up***	*n* (%)	*n* (%)	0.05
Only MMF	1 (7.7%)	34 (41%)	
Only SR	5 (38.5%)	26 (31.3%)	
SR after MMF failure	7 (53.8%)	23 (27.7%)	
***Therapy duration (months)***	Median, IQR	Median, IQR	
Total	54 (55)	47 (59.5)	0.337
Mycophenolate	20 (62.5)	32.5 (53.8)	0.925
Sirolimus	40 (46.8)	23 (44)	0.107

ALPS, autoimmune lymphoproliferative syndrome; IQR, interquartile range; MMF, mycophenolate mofetil; SIE, severe infectious event; SR, sirolimus.

**Table 2:** Clinical features of patients with severe infectious events.

Patient	Phenotype	Genetic defect	SIE (*n*)	MMF (months)	SR (months)	Type of infection	Previous therapy	Treatments during SIE
1	ALPS-like	–	2	53	31	-Pyelonephritis -Sepsis by *E. coli*	MTX, HCQ, azathioprine, CPM, tacrolimus, thalidomide, bortezomib, eculizumab, belimumab MMF	SR
2	ALPS	–	1	144	0	Pneumonia	–	MMF
3	ALPS	–	1	0	22	Gastroenteritis	–	SR
4	ITP	–	1	4	46	Pneumonia	–	SR
5	ALPS-like	–	2	0	6	-Pneumonia -Sepsis	Steroid	SR
6	ALPS-like	NEMO	1	0	72	Pneumonia	Steroid	SR
7	ALPS-like	LRBA	1	24	100	Pneumonia	Steroid, Abatacept	SR
8	ALPS-like	IPEX	1	15	73	Pneumonia	–	SR
9	ALPS-like	–	1	6	52	SARS-CoV-2 Pneumonia	Steroid, IVIG	SR, steroid thalidomide
10	ALPS-like	RMRP	2	0	98	-Abdominal infection -Meningitis (Cryptococcus)	IVIG	SR
11	ALPS-like	STAT3 GOF	1	11	40	Pneumonia	Steroid, IVIG, rituximab	SR
12	ALPS-like	–	1	0	35	Pneumonia	Steroid	SR
13	ALPS-like	TNFSR3B	1	1	4	Viral pneumonia	–	SR

ALPS, autoimmune lymphoproliferative syndrome; CPM, cyclophosphamide; ES, Evans syndrome; HCQ, hydroxychloroquine; IPEX, immune dysregulation, polyendocrinopathy, enteropathy, X-linked; ITP, immune thrombocytopenia; LRBA, lipopolysaccharide-responsive and beige-like anchor protein; MMF, mycophenolate mofetil; MTX, methotrexate; NEMO, NF-kb essential modulator; RMRP, RNA component of the mitochondrial RNA-processing endoribonuclease; SIE, severe infectious event; SR, sirolimus; STAT3, signal transducer and activator of transcription 3.

**Table 3:** SIE incidence rate divided by diagnosis.

	Patients (*n*)	Months of FU (total)	SIE (*n*)	SIE (incidence *100 p/m/r)	95% CI	Cumulative incidence (%)
Global cohort						
***Diagnosis***						
ALPS-like	47	3,132	13	0.42	0.24–0.71	27.66
Other AICs	49	3,488	3	0.09	0.03–0.27	6.12
Overall	96	6,620	16	0.24	0.14–0.39	16.67
***Treatment***						
MMF	35	2,494	1	0.04	0.01–0.28	2.86
SR	33	2,091	9	0.43	0.22–0.83	27.27
Sequential MMF+SR	28	2,035	6	0.29	0.13–0.66	21.43
Patients diagnosed after 2015						
***Diagnosis***						
ALPS-like	22	1,181	5	0.42	0.18–1.02	22.73
Other AICs	27	1,685	1	0.06	0.01–0.42	3.7
Overall	49	2,866	6	0.21	0.09–0.47	12.24
***Treatment***		**Months of therapy** **(total)**				
MMF	16	573	0	0.00		0
SR	17	558	3	0.54	0.17–1.67	17.65
Sequential MMF+SR	16	490	3	0.61	0.20–1.90	18.75

**Figure 2:**
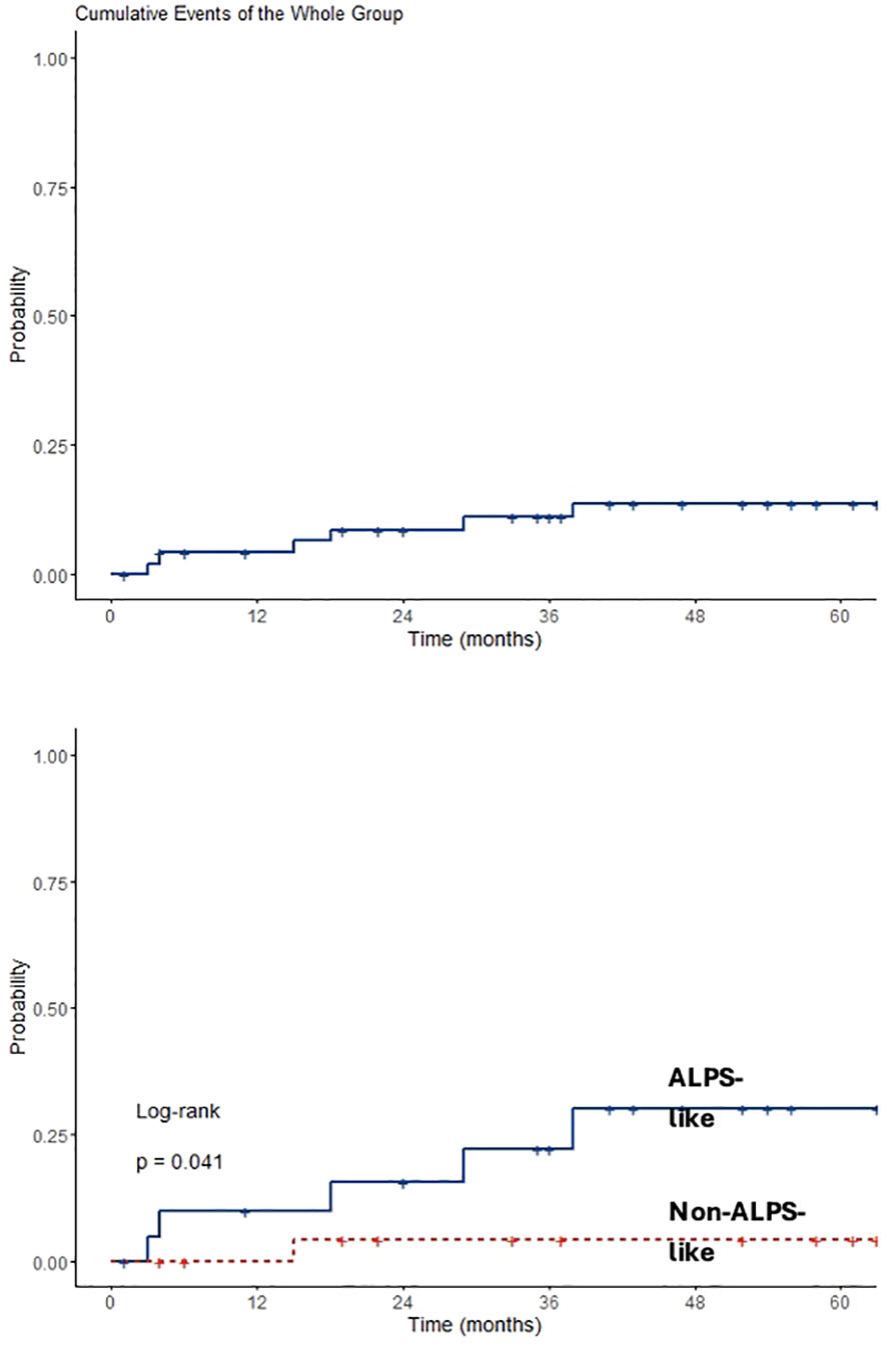
Overall and diagnosis-based SIEs cumulative risk.

Analyzing data only from 49 patients diagnosed after 2015, an overall observation time of 2,866 months was recorded. ALPS-like and other AIC patients were followed for a total of 1,181 and 1,685 months, respectively. MMF and SR alone treatments were given for 573 and 558 months, respectively. Sequential MMF/SR therapies covered a period of 490 months. Six SIEs occurred in 6/49 patients (12%), with a crude rate of 0.21 *100 p/m/r (95% CI 0.08–0.5). Also in this case, a statistically significant difference was found between ALPS-like and other AICs (0.42 versus 0.06 *100 p/m/r; *p* = 0.03). All episodes occurred during SR treatment. Among them, 3/6 (50%) received SR treatment after MMF failure. Data are summarized in **Table 3**. Overall SIE cumulative hazard of this cohort was 18.6% at 60 months (95% CI 3.4–31.4). In this group of patients, a higher cumulative risk of SIE after 5 years was found between ALPS-like patients (26.1%; 95% CI 3.2–43.5) and other AICs (4%; 95% CI 0–11.4; *p* = 0.041) (**Figure 2**).

## Discussion

Over the last decade, efficacy and safety of SR and mycophenolate therapy in patients with ALPS, ALPS-like, and other AICs have been described in several reports (1, 2, 5–7, 9, 12, 15–17, 21–33). However, there are no studies reporting the actual risks of infections in patients undergoing these treatments over the years. To the best of our knowledge, this is the largest cohort of patients in which infection rate and SIEs have been quantitatively evaluated and shows that only 13% of patients had severe events requiring hospitalization and/or intravenous antibacterial therapy.

In our records, only one patient treated with MMF between 2015 and 2023 developed SIEs, which is in line with previous data (29, 32–34). In the pediatric setting, some reports on children treated with MMF for non-hematological diseases showed similar results (34, 35), but no data were available on hematological patients apart from two case reports (31, 36).

In our cohort, SIEs occurred in all cases but one under SR treatment. Very few reports have shown the relationship between SR therapy and infections in hematological patients and no increase was observed in infection frequency or severity (21, 30). In the few articles on infection rate in patients with PIK3CA-related overgrowth spectrum (PROS) treated with SR, a possible increased risk of infections was highlighted, which was similar to what was reported in our cohort (41% vs. 47%) (37, 38). However, that report was not focused on SIEs, which are more clinically relevant. Indeed, in our study, we analyzed SIEs over a long follow-up and demonstrate that they occur quite rarely (13%). Moreover, unlike PROS, the analysis of our patients with SIEs excluded the first 4 weeks of treatments when any possible event would be hardly related to the immunosuppressive effect that takes 6–8 weeks to become fully established (37, 38). In our cohort, most patients developing SIEs under SR were affected with ALPS-like syndromes. Compared to isolated AIC, which in our cohort was represented only by primary ITP, and ALPS subjects, it is known that patients affected with these disorders are known to have an increased risk of developing infections, mostly related to lymphopenia and hypogammaglobulinemia, which were present in all of our cases. In addition, our ALPS-like patients developing SIEs were in most cases affected with underlying genetic disorders as the STAT3 GOF syndrome, TACI, or NEMO deficiency that are, per se, typically associated with a high infection risk (39–41). Similarly, all our patients with a diagnosis of Cartilage-Hair-Hypoplasia (CHH), IPEX, and LRBA deficiency developed a complex phenotype with autoimmunity and immunodeficiency (42, 43). Of note, the ALPS-like subgroup, in addition to carrying the highest SIE rate, was also the one in which genetic disorders were more frequently detected. This highlights the importance of specific genetic diagnosis to also evaluate the infection risk. Interestingly, the higher occurrence of SIEs during SR rather than during MMF seems in contrast with the knowledge that SR has a prevalent immunoregulatory than immunosuppressive effect. A possible explanation could reside in the higher number and in the longer duration of previous therapies received by this subgroup of patients.

In conclusion, still with the limitations of a retrospective data review, our findings suggest that MMF and SR treatment is associated with a very limited risk of SIEs in patients with AICs and PIRDs and that this risk is mainly related to the immunodeficient status related to the underlying ALPS-like disorder. Nevertheless, prospective clinical trials are needed to confirm these results.

## Data availability statement

The raw data supporting the conclusions of this article will be made available by the authors, without undue reservation.

## Ethics statement

The studies involving humans were approved by Comitato Etico Regione Liguria. The studies were conducted in accordance with the local legislation and institutional requirements. Written informed consent for participation in this study was provided by the participants’ legal guardians/next of kin.

## Author contributions

MC: Data curation, Formal analysis, Investigation, Methodology, Visualization, Writing – original draft, Writing – review & editing. EP: Conceptualization, Data curation, Formal analysis, Investigation, Project administration, Visualization, Writing – original draft, Writing – review & editing, Methodology, Supervision, Validation. MMa: Data curation, Formal analysis, Investigation, Methodology, Visualization, Writing – original draft, Writing – review & editing. GD’O: Data curation, Investigation, Writing – review & editing. MLi: Writing – review & editing, Investigation, Methodology. MG: Investigation, Validation, Writing – review & editing. LA: Investigation, Writing – review & editing. SP: Investigation, Writing – review & editing. AG: Investigation, Data curation, Methodology, Writing – review & editing. MLa: Investigation, Methodology, Writing – review & editing. GB: Data curation, Investigation, Writing – review & editing. DG: Investigation, Writing – review & editing. GR: Project administration, Supervision, Writing – review & editing. CD: Project administration, Supervision, Writing – review & editing. FF: Project administration, Supervision, Writing – review & editing. EC: Project administration, Supervision, Writing – review & editing. MMi: Conceptualization, Data curation, Formal analysis, Investigation, Methodology, Project administration, Resources, Supervision, Validation, Visualization, Writing – original draft, Writing – review & editing.
